# Site of relapse of ductal adenocarcinoma of the pancreas affects survival after multimodal therapy

**DOI:** 10.1186/s12893-021-01082-w

**Published:** 2021-03-03

**Authors:** S. A. Safi, N. Lehwald-Tywuschik, A. Rehders, G. Fluegen, L. Haeberle, V. Keitel, W. T. Knoefel

**Affiliations:** 1 Department of Surgery (A), Medical Faculty, Heinrich-Heine-University and University Hospital, Duesseldorf, Germany; 2 Institute of Pathology, Medical Faculty, Heinrich-Heine-University and University Hospital, Duesseldorf, Germany; 3 Department of Gastroenterology, Hepatology and Infectious Diseases, Medical Faculty, Heinrich-Heine-University and University Hospital, Duesseldorf, Germany

**Keywords:** PDAC, Ductal adenocarcinoma of the pancreas, Oligometastatic, Survival outcome, Pulmonary metastases, Local recurrence, Hepatic metastases

## Abstract

**Background:**

Ductal adenocarcinoma of the pancreas (PDAC) remains one of the most lethal malignancies. To date, no guidelines exists for isolated resectable metachronous disease. It is still unknown, which patients may benefit from relapse surgery. The aim of our study was to compare disease free survival (DFS) and post relapse survival (PRS) in patients with isolated local recurrence, metachronous hepatic or pulmonary metastases.

**Methods:**

Patients with isolated resectable local recurrence, metachronous hepatic or pulmonary metastases were included for survival analyses. PRS of surgically treated patients (local (n = 11), hepatic (n = 6) and pulmonary metastases (n = 9)) was compared to conservatively treated patients (local (n = 17), hepatic (n = 37) and pulmonary metastases (n = 8)).

**Results:**

Resected PDAC patients suffering from isolated metachronous hepatic metastases initially had a higher T-stage and venous invasion (V1) compared to the other patients. DFS in the metachronous pulmonary metastases group was longer compared to DFS of the hepatic metastases and local recurrence groups. Surgical resection significantly improved PRS in patients with local recurrence and pulmonary metastases, when compared to patients receiving chemotherapy alone. Very-long term survivors (> 5 years) were detected following secondary resection of local recurrence and 45% of these patients were still alive at the end of our study period.

**Conclusion:**

Although DFS in PDAC patients suffering from isolated local recurrence was dismal and comparable to that of patients with isolated hepatic metastases, very-long term survivors were present only in this group. These results indicate that a surgical approach for isolated local recurrence, if anatomically possible, should be considered.

## Background

The ductal adenocarcinoma of the pancreas (PDAC) has a dismal prognosis and the median overall survival of ~ 6 months did not improve over the past decade. Thus, oncological advances in the treatment of PDAC have been slow [1]. By 2030, it is estimated to become the second leading cause of cancer-related death in the United States and also in Germany [2, 3]. To date, the only curative therapy remains the margin-negative oncological resection in combination with an adjuvant treatment regime, starting within 6 weeks after the operation [4, 5].

Most PDAC patients suffer from peritoneal, hepatic or pulmonary metastases [6]. At diagnosis, 50% of the patients present with synchronous metastases and further 30% present with locally advanced disease, which are not suitable for surgery. Thus, only 20% of the patients suffering from PDAC receive surgery with curative intent. Therefore, PDAC is still regarded as one of the most lethal cancers, also indicated by a very high mortality-to-incidence ratio [1, 7].

Following international guidelines, upfront surgery and adjuvant chemotherapy for patients with localized and resectable PDAC are indispensable in the treatment of this disease [8]. However, no guidelines exist for the treatment of metachronous PDAC metastases and treatment options are individualized due to a lack of clinical investigation. Decision making is dependent on the patients’ performance status, staging of the primary tumor, tumor load and location of metastases and disease free survival. One possibility to stratify patients for an individualized treatment regime is the genomic sequencing of primary tumor and metastases. Following the success of similar standardized genomic approaches in other tumor entities, this targeted approach is going to be of interest in PDAC treatment in the future [9–11].

Chemotherapy with palliative intent or best supportive care is still the standard treatment for patients with synchronously metastasized PDAC [12, 13]. To date, no standardized surgical treatment exists for patients with metachronous isolated disease. Therefore, in current clinical practice, unlike in other malignancies, metachronous metastasectomy has barely been performed for PDAC. In these metachronous patients, chemotherapy largely remains the only treatment option on offer. Therapeutic regimes, such as FOLFIRINOX (folinic acid, fluorouracil, irinotecan, oxaliplatin), have very recently been established as primary treatment option and most patients with an adequate clinical condition can tolerate these cytotoxic regimes [13, 14]. To date, it remains uncertain which patients may benefit from surgical metastasis resection. Yet, whether chemotherapy-naive patients with resectable relapse burden, patients with a stable relapse or patients with relapse regression following chemotherapy will benefit from a surgical approach, is hardly investigated.

The aim of our study was to analyze clinical outcomes of patients who received surgery in our department for isolated pulmonary or hepatic metastases, or local recurrence, and to compare these to patients treated with conservative therapy for similar metastasized disease.

## Methods

### Patient selection and clinicopathological data

Patients suffering from ductal adenocarcinoma of the pancreas and consecutively received surgery between September 2003 and May 2020 at the Heinrich Heine University Hospital of Duesseldorf were included in the study. Exclusion criteria were: (1) malignancies of the pancreas other than ductal adenocarcinoma, (2) TNM staging lacking information on lymphatic, perineural and venous invasion (Lx, Pnx, Vx), (3) UICC stage IV resected PDACs, (4) patients with insufficient follow-up information, (5) primary palliative treatment and (6) patients who succumbed within 30-days of the operation. Clinical data of these consecutively treated patients collected from patient’s medical records was compiled into an Excel®-file database and analyzed retrospectively.

The TNM staging (size of tumor / involvement of adjacent arteries, lymph node status and status on distant metastasis), along with grading, perineural invasion, lymphatic and venous invasion was retrospectively collected from the original histopathological reports for each patient. The TNM staging was updated to the 8th Edition of the UICC TNM classification of malignant tumors, where applicable [17]. Clinicopathological data (gender, age at the time of surgery, overall survival (OS) and results of follow-up examinations including time and site of metastases) were retrieved. In order to evaluate disease free survival (DFS), all lesions of suspected metachronous disease detected by any diagnostic imaging were included into the survival analysis. Furthermore, post relapse survival (PRS), survival from date of relapse diagnosis until death, was calculated.

Patients suffering from isolated and resectable metastases were selected from the total study cohort and included in this analysis. Only cases in which a surgically resectable relapse was diagnosed singularly in either one of the three studied compartments (liver, lung, local) were included for analysis. Patients with diffuse metastases not eligible for surgery, poor ECOG performance score, palliative patients and patients lost to follow-up were excluded. Only patients who received surgery or chemotherapeutic therapy for single site relapse were identified and included. In the chemotherapeutic therapy group, only patients with a homogenous distribution of amount, size and location of relapse were included.

Six groups were generated for the analysis of metachronous disease: patients receiving individualized surgery for either (1) local recurrence (surglocal), (2) hepatic (surghep) or (3) pulmonary (surgpul) metastases; patients who received only chemotherapy for (4) local recurrence (chemlocal) or metachronous (5) hepatic (chemhep) or (6) pulmonary (chempul) metastases.

The analysis was performed in conformity to the Declaration of Helsinki and to good Clinical Practice. Furthermore, a positive vote of the Institutional Review Board (IRB) of the Ethics Committee, Heinrich Heine University Duesseldorf (IRB-no. 2019–473-2), was retrieved.

### Statistical analysis

The Wilcoxon test was used to analyze differences in clinicopathological data between the six subgroups. The Mann–Whitney U test was used to examine numerical data and to correlate between clinicopathological variables. For categorical data, the chi-square test was applied. DFS and PRS were included for outcome measures. Disease free survival described the period from the date of primary surgery until the date of diagnosed metachronous metastases or local recurrence. Post relapse survival included the period between relapse diagnoses and death or last follow-up. Kaplan–Meier curves were generated and analyzed using the log-rank (Mantel Cox) test. Analyses were performed using SPSS® statistics for Windows (version 25.0; SPSS, Inc., Chicago, IL, USA). P < 0.05 was considered to indicate a statistically significant difference.

## Results

### Clinicopathological data for metachronous disease

Out of the 346 primarily operated patients, 141 patients met our pre-defined inclusion criteria for analysis of isolated resectable metachronous disease during follow-up. The included patients had all received relapse therapy between 2007 and 2016 (Table 1). In 128 patients, the primary PDAC was located in the pancreatic head (90.8%) while in 13 patients (9.2%) the tumor originated from the pancreatic tail (Table 1). In our total cohort of patients, the mean follow-up period was 49.65 months (95%CI: 39.74–59.55 months). The median age of all 141 patients at the time of primary surgery was 67 years (range 17–95 years). Our collective consisted of 76 males (53.9%) and 65 females (46.1%). In correlation analysis of TNM staging of the primary tumor and site of metachronous disease, T- and N-stage, tumor grading, positive perineural and lymphatic invasion did not correlate with site of metachronous disease. However, patients with positive venous invasion (V1) later succumbed to metachronous hepatic metastases (Table 1).

**Table 1:** Demographic table of study collective and correlation analysis between relapse compartment and clinicopathological variables from primary PDAC; n = 141

	**No metastases** n = 54	**Hepatic, **n = 43	**Pulmonary, **n = 17	**Local, **n = 28	***p-value***
Age in years					
Median (range)	68 (17–95)	69 (46–86)	64 (41–81)	63.5 (42–88)	*0.521*
Gender					*0.109*
Female	27	19	8	11	
Male	27	24	9	17	
Tumor localisation					*0.265*
Pancreatic head	45	42	16	25	
Pancreatic tail	9	1	1	3	
T-stage					*0.478*
T1	8	2	1	2	
T2	26	27	9	20	
T3	20	13	7	5	
T4	0	1	0	1	
N-stage					*0.157*
N0	17	6	3	6	
N1	33	31	14	22	
N2	4	6	0	0	
Grading					*0.446*
G1/G2	33	25	8	19	
G3	21	18	9	9	
Pn					*0.483*
Pn0	15	7	5	5	
Pn1	36	33	12	20	
Missing	3	3	0	3	
L					*0.161*
L0	34	20	6	14	
L1	17	20	11	11	
Missing	3	3	0	3	
V					*0.011*
V0	41	23	15	22	
V1	10	17	2	3	
Missing	3	3	0	3	
Resection status					*0.305*
R0CRM−	49	39	14	21	
R1/R0CRM +	5	4	3	7	
Chemotherapy					*0.692*
MD regime	14	8	3	7	
FOLFIRINOX	3	6	1	1	
Gemcitabine mono	30	29	9	15	
No CTx	7	0	4	5	

Only venous invasion correlated with metachronous hepatic metastases. Pearson Test and Mann–Whitney U test were used to test for statistical significance. p-value ≤ *0.05* indicates significance

*CTx* chemotherapy*, MD* multidrug*, Pn* perineural invasion*, L: lymphatic invasio, V venous invasion*

### Relapse surgery and secondary chemotherapy initiation:

Out of the total study collective, 54 patients did not succumb to metachronous relapse in the study period. In the remaining cohort, 28 patients received therapy for isolated local recurrence (group surglocal n = 11; chemlocal n = 17). Further 17 patients were treated for isolated metachronous pulmonary metastases (group surgpul n = 9; chempul n = 8) and 43 patients received therapy for isolated metachronous hepatic metastases (group surghep n = 6; chemhep n = 37) (Table 2).

**Table 2:** Correlation analysis between relapse compartment and survival time stratified according to secondary therapy; n = 87

Recurrence/metastases	**Total**		**Surgery**		**Chemotherapy**		*p-value* *0.000*
	**n**	**%**	**n**	**%**	**n**	**%**	
Hepatic	43		6		37		
Local	28		11		17		
Pulmonary	17		9		8		

Relapse Chemotherapy/procedure	**Hepatic, n**	**Local, n**	**Pulmonary, n**	*p-value* *0.900*
Gemcitabine	27	12	5	
MD regime	10	5	3	
FOLFIRINOX	–	–	–	
BSC	–	–	–	

Fisher exact test was used to test for statistical significance. p-value ≤ *0.05* indicates significance

*BSC* best supportive care*, MD* multi-drug

**Fig. 1:**
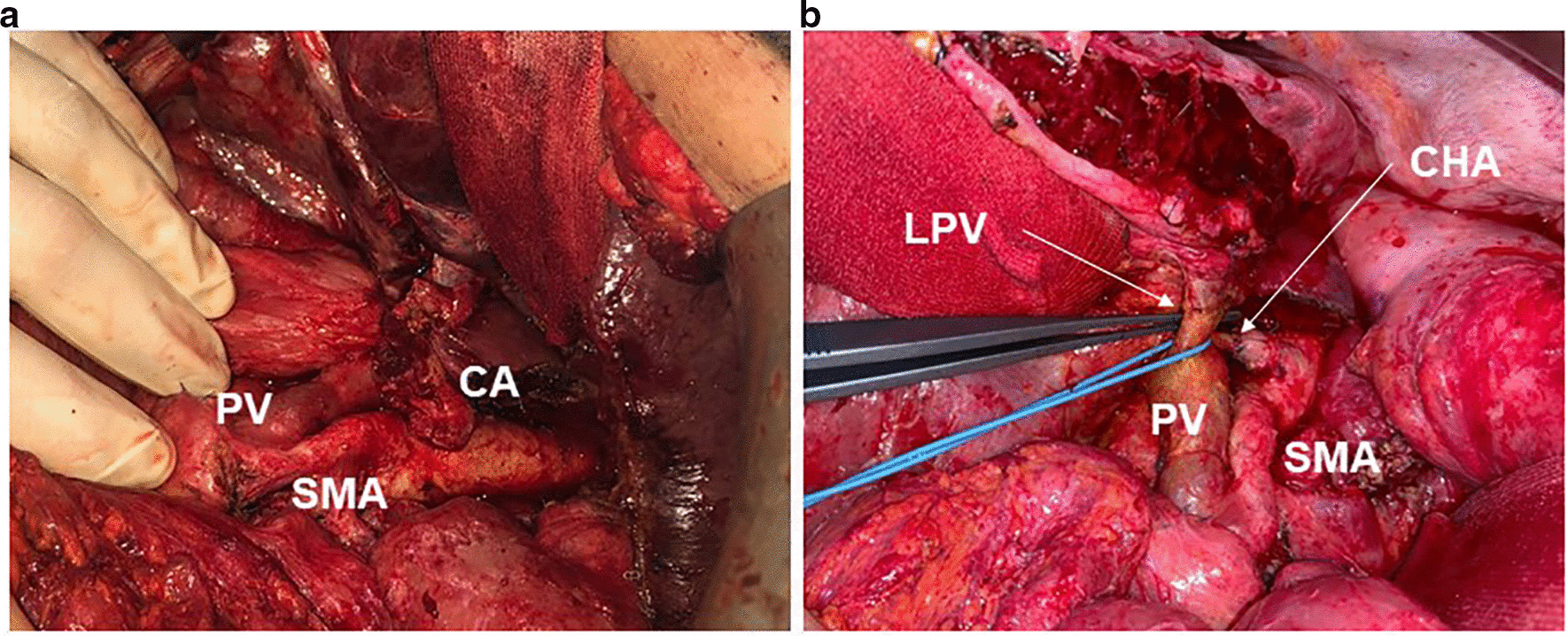
Intraoperative view **a** before resection and **b** after resection of local recurrence. The tumor was infiltrating the left portal vein and left hepatic artery. Tumor resection was performed with synchronous left lateral hemihepatectomy and resection/reconstruction of the left portal vein and resection of the left hepatic artery with reconstruction of the common hepatic artery. Primary tumor staging pT2 pN1 (3/56) L0 V0 Pn1 G2 R0CRM + (CHA: common hepatic artery, LPV: left portal vein, PV: portal vein, SMA: superior mesenteric artery)

For hepatic metastases, one right hemi-hepatectomy and five atypical non-anatomical resections were performed. For pulmonary metastases, patients received four right and five left atypical resections via video-assisted thoracoscopy (VATS). For local recurrence, one gastrectomy with atypical resection of the left diaphragm, nine salvage-pancreatectomies with simultaneous right hemicolectomies and one partial psoas muscle resection were performed (Fig. 1, Table 2). In all 28 patients, margin negative resections were achieved. Out of the 59 conservatively treated patients, 44 patients (74.6%) received gemcitabine mono, while a combination therapy with gemcitabine was administered to 15 patients (25.4%). None of the patients received FOLFIRINOX as a secondary therapy line. The distribution of secondary conservative treatment regimens between the isolated relapse compartments was homogenous and without statistical significance (*p* = *0.900*) (Table 2).

### Disease free survival and post relapse survival:

**Fig. 2:**
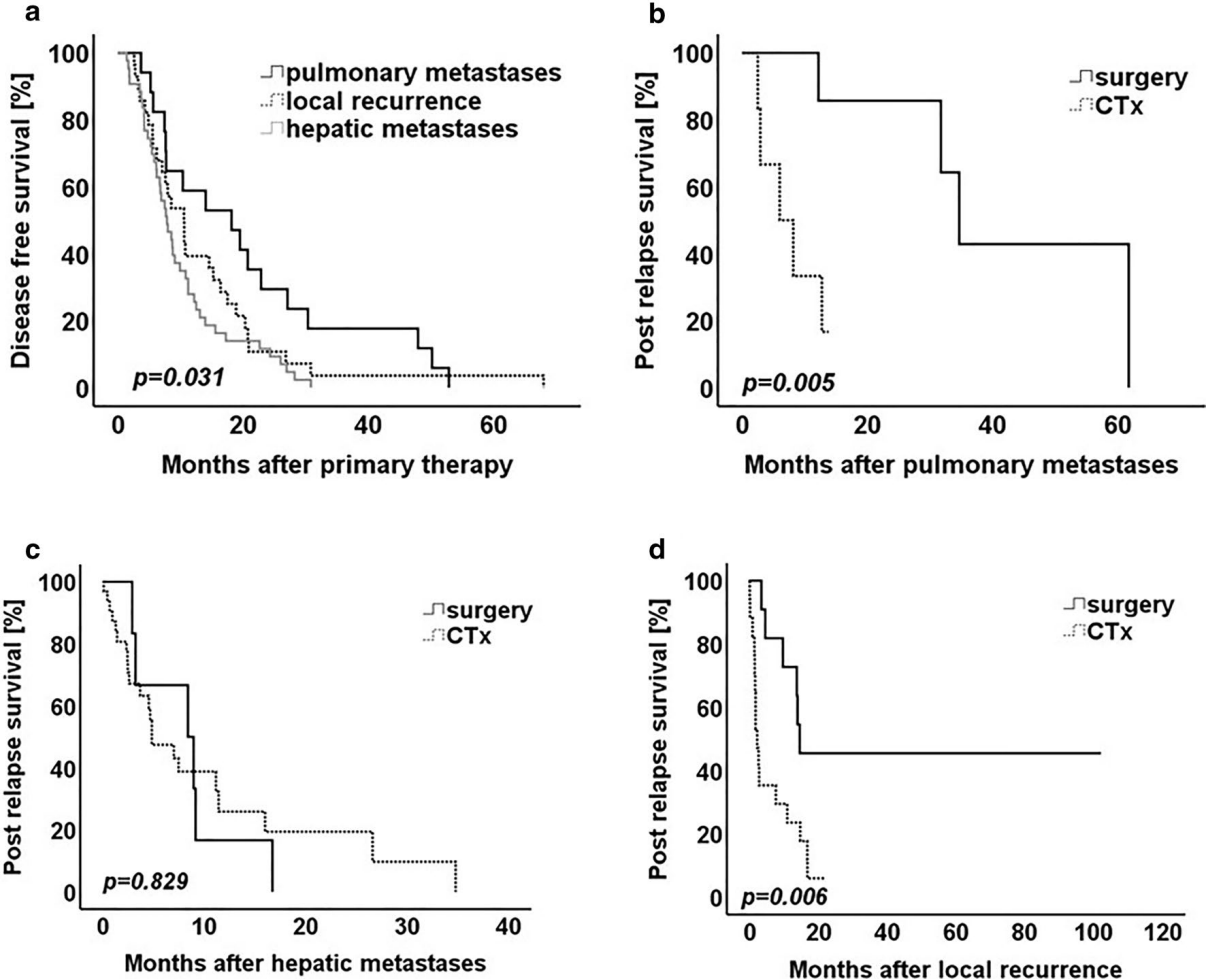
Kaplan–Meier curve for **a** disease free survival of patients dependent on the metastatic location. **b** Post relapse survival of patients with pulmonary metastases. **c** Post relapse survival of patients with hepatic metastases. **d** Post relapse survival of patients with local recurrence. Log rank test was used to test for significance. p-value ≤ *0.05* indicates significance

Median survival data is summarized in Table 3. The median disease free survival of all 141 patients was 13.09 months (95%CI 9.46–16.72 months). The median disease free survival in the 87 patients with isolated relapse was 13.28 months (95%CI 10.74–15.83 months). In patients in the pulmonary metastasis group, the median DFS of 18.15 months was longer, compared to the DFS of patients in the local recurrence and hepatic metastasis groups (*p* = *0.031*) (Table 3, Fig. 2a). The median DFS of 10.55 months in the local recurrence group was similar to the median DFS of 7.83 months in hepatic metastasis group (*p* = *0.180*) (Table 3, Fig. 2a).

**Table 3:** Survival data stratified according to relapse location and treatment modality; n = 87

	DFS before 2^nd^ treatment		A	DFS before surgery		A	RSS after surgery	
			B	DFS before chemotherapy		B	RSS after chemotherapy	
	Median (months)	95%CI		Median (months)	95%CI		Median (months)	95%CI
Pulmonary	18.15	0.30–35.99	A	22.85	14.10–31.64	A	61.71	–
			B	7.63	4.76–10.49	B	5.99	0.0–12.3
			p-value		*0.012*	p-value		*0.005*
Hepatic	7.83	5.76–9.89	A	7.47	0.00–15.56	A	8.39	1.52–15.26
			B	7.83	5.75–9.90	B	4.84	1.25–8.42
			p-value		*0.897*	p-value		*0.829*
Local	10.55	7.14–13.96	A	10.75	2.30–19.22	A	51.85	24.65–79.10
			B	7.95	2.10–13.84	B	6.20	3.41–9.89
			p-value		*0.972*	p-value		*0.006*

Patients with isolated pulmonary metastases showed the best DFS. Surgery significantly influenced PRS in patients with isolated pulmonary metastases and local recurrence. Log rank test was used to test for statistical significance. p-value ≤ 0.05 indicates significance

*CI* confidence interval,* DFS* disease free survival,* RSS* relapse specific survival, 2^*nd*^* treatment* relapse surgery or relapse chemotherapy

To assess post relapse survival, 54 patients without metachronous relapse were removed for further survival analysis. In order to exclude selection bias and differences in tumor biology between the applied treatment modalities for metachronous disease, DFS before secondary relapse treatment initiation was stratified according to the treatment modality applied (Table 3). The median DFS before secondary therapy initiation was similar in patients with isolated hepatic metastases and isolated local recurrence between both treatment modalities (surgery and chemotherapy) (*p* = *0.897* for surghep vs. chemhep *and p* = *0.972* for surglocal vs. chemlocal) (Table 3). However, DFS in patients in the resected pulmonary metastasis group was significantly longer when compared to patients who only received chemotherapy (*p* = *0.012*) (Table 3). Hence, a selection bias was detected only in patients with isolated pulmonary metastases.

To evaluate the post relapse survival benefit after surgery or conservative treatment, the post relapse survival for each analyzed relapse compartment was analyzed after therapy initiation**.** Surgical therapy significantly improved survival in patients with isolated local recurrence and pulmonary metastases, when compared to chemotherapy (*p* = *0.009* for surgpul vs. chempul; *p* = *0.006* for surglocal vs. chemlocal and *p* = *0.829* for surghep vs. chemhep) (Fig. 2b–d). Although the median PRS was similar in the pulmonary metastasis and local recurrence groups, all patients suffering from isolated pulmonary metastases succumbed after secondary therapy in our study (Table 3, Fig. 2b). Five of the 11 patients with surgically resected isolated local recurrence were still alive 102 months after relapse surgery. In spite of a dismal median DFS of patients in the local recurrence group, which was similar to the DFS of patients with hepatic metastases, all very long-term survivors (> 5 years) of this cohort were found only in the group of patients with surgically resected isolated local recurrence (Fig. 2d).

## Discussion

To date, the survival outcome of metachronous metastasized PDAC has never been evaluated in regard to the different relapse compartments (local, hepatic, pulmonary) in one single centre cohort. In previous studies, only one specific relapse compartment was investigated [15–18]. In our study, we could show for the first time that DFS was longer in patients with isolated pulmonary metastases when compared to patients with metachronous hepatic metastases and local recurrence. Relapse surgery significantly improved survival of patients with isolated pulmonary metastases and local recurrence, when compared to patients treated only with chemotherapy. Long-term survivors (> 5 years) were, however, only found in patients receiving surgery for local recurrence, despite the dismal disease free survival of this subgroup.

Over the past decade, the role of metastasectomy for localized pulmonary metastases in PDAC was elucidated. It was reported that long-term survivors of PDAC and patients with a superior DFS were often the patients suffering from isolated pulmonary disease. Our findings are in-line with these previous observations [15, 16, 19]. The DFS of the isolated pulmonary metastasis group was significantly longer, compared to the hepatic metastasis and local recurrence groups. Survival before diagnosis of the local recurrence and hepatic metastasis groups was comparably poor. However, interestingly, survival following surgery for isolated local recurrence and pulmonary metastases were similar and, importantly, in both cases longer, compared to patients following surgery for hepatic metastases. Very-long-term survivors were only found in the subgroup receiving surgery for local recurrence. Thus, local recurrence presumably reflected insufficient tumor clearance during primary surgery in a tumor with a less adverse systemic cancer biology [20]. This indicates that a surgical approach for local recurrence is feasible, if resectability is provided as described by Strobel et al.[17]. Thus, even a very early diagnosis of local relapse should not preclude relapse surgery, as long term survival seems still possible.

In two recent meta-analyses from 2017 by Groot et al. and from 2019 by Moletta et al., the authors investigated the survival difference by different therapeutic approaches for local recurrence [21, 22]. In summary, both analyses showed that surgery is feasible and does prolong survival. However, both meta-analyses showed a certain degree of heterogeneity due to multiple interdisciplinary approaches. In some studies, adjuvant therapy was administered after the initial operation [23]. Others have offered adjuvant therapy after both initial and relapse operation [18, 24–27]. Neoadjuvant therapy prior to the relapse operation was only applied in two studies [17, 28]. Our data clearly revealed, if resectability is provided, upfront relapse surgery in patients with isolated local recurrence after multimodal therapy does significantly prolong survival, compared to patients who received chemotherapy alone. 45% of all patients receiving surgery for isolated local recurrence were still alive at the conclusion of our study period and presented with no further relapse in follow-up investigations over a duration of 102 months. Hence, this excellent survival outcome presumably reflects a less adverse tumor biology in this subgroup despite the relatively short DFS. In conclusion, this emphasizes a primary radical degree of surgery in order to prevent margin positive resections and thus local relapse in follow-up, with a further burden of surgical re-exploration [29, 30].

Since the DFS of the surgery subgroup of the pulmonary metastasis group was significantly longer compared to the chemotherapy alone subgroup, our study was limited in terms of selection bias. Thus, the post relapse survival after surgery for isolated pulmonary metastases has to be considered with certain precautions. However, it is a well-established observation that a superior DFS is mostly found in patients with pulmonary metastases [19, 31–33]. This is in-line with a recent SEER analysis from *Liu *et al., who demonstrated that resection in highly selected patients with either isolated synchronous or metachronous pulmonary metastases is correlated with a significant survival benefit in PDAC [34]. Furthermore, no advice can be given on the setting of multimodality for metachronous disease (preoperative vs. postoperative vs. perioperative), as all patients received upfront surgery with adjuvant therapy compared to patients with only chemotherapy.

Isolated resectable relapse is rare in patients with prior therapy for PDAC. Thus, no larger case series exist in the literature and randomized control studies are not available. In selected patients, however, we and others have shown that metastasis surgery followed by adjuvant chemotherapy is feasible [17].

One weakness of this study is the relatively prolonged study period of 9 years. Hence, during this time, adjuvant treatment protocols following primary surgery have changed. However, due to our focus on post relapse survival, it seems reasonable to assume that an adjuvant treatment following the primary surgery only has a limited effect on the survival after relapse diagnosis and treatment. To date, no multicenter studies exist which elucidate the survival effect of FOLFIRINOX in patients with metachronous relapse. For this matter, future multicenter studies are needed to clarify the impact of different chemotherapeutic therapies on survival outcome. To further select patients who most likely benefit from relapse surgery or chemotherapy for prolonged survival, enhanced preoperative diagnostics are clearly needed. Thus, future studies should investigate the genomic information of the primary tumor and the metastasis to detect mutational changes for targeted therapies. Furthermore, sensitive detection methods to identify circulating tumor cells to estimate the presumed tumor load and identify auxiliary genomic subgroups should be evaluated and might help to detect patients at increased risk of relapse [35].

## Conclusion

In summary, patients suffering from resectable isolated local recurrence potentially benefit from relapse surgery despite a dismal DFS. Very long term survivors were only found in this subgroup of patients. Thus, a dismal DFS should still encourage surgical exploration if resectability is provided. However, DFS of patients suffering from isolated pulmonary metastases was longer, compared to the other patient groups, and surgical resection of isolated pulmonary metastases should also be considered. Patient selection is indispensable, as long-term survival is only achievable by an adequate multimodal therapy approach. In order to provide a clear path for clinical decision making when faced with isolated PDAC metastasis, prospective multi-center trials investigating multimodal therapies in large cohorts are clearly warranted [5, 36–38].

## Data Availability

The datasets used and/or analyzed during the current study are available from the corresponding author on reasonable request.

## Abbreviations

Chem: Chemotherapy
CI: Confidence interval
CTX: Chemotherapy
DFS: Disease free survival
FOLFIRINOX: Folinic acid, fluororuracil, irinotecan, oxaliplatin,
Hep: Hepatic
HR: Hazard ratio
Meta: Metachronous/metachronously
OS: Overall survival
Pall: Palliative
PDAC: Pancreatic ductal adenocarcinoma
PRS: Post relapse survival
Pul: Pulmonary
Surg: Surgical
syn: Synchronous/synchronously
UICC: Union for international cancer control
